# Statistical Analysis of 3D Images Detects Regular Spatial Distributions of Centromeres and Chromocenters in Animal and Plant Nuclei

**DOI:** 10.1371/journal.pcbi.1000853

**Published:** 2010-07-08

**Authors:** Philippe Andrey, Kiên Kiêu, Clémence Kress, Gaëtan Lehmann, Leïla Tirichine, Zichuan Liu, Eric Biot, Pierre-Gaël Adenot, Cathy Hue-Beauvais, Nicole Houba-Hérin, Véronique Duranthon, Eve Devinoy, Nathalie Beaujean, Valérie Gaudin, Yves Maurin, Pascale Debey

**Affiliations:** 1 INRA, UMR1197 Neurobiologie de l'Olfaction et de la Prise Alimentaire, Jouy-en-Josas, France; 2 Université Paris-Sud 11, UMR 1197, Orsay, France; 3 IFR144 Neuro-Sud Paris, France; 4 UPMC, Université Paris 06, France; 5 INRA, UR341, Mathématiques et Informatique Appliquées, Jouy-en-Josas, France; 6 INRA, UR1196 Génomique et Physiologie de la Lactation, Jouy-en-Josas, France; 7 INRA, UMR1198 Biologie du Développement et Reproduction, Jouy-en-Josas, France; 8 ENVA, Maisons Alfort, France; 9 INRA, Institut J.-P. Bourgin, UMR1318 INRA-AgroParisTech, Versailles, France; Institut Pasteur, France

## Abstract

In eukaryotes, the interphase nucleus is organized in morphologically and/or functionally distinct nuclear “compartments”. Numerous studies highlight functional relationships between the spatial organization of the nucleus and gene regulation. This raises the question of whether nuclear organization principles exist and, if so, whether they are identical in the animal and plant kingdoms. We addressed this issue through the investigation of the three-dimensional distribution of the centromeres and chromocenters. We investigated five very diverse populations of interphase nuclei at different differentiation stages in their physiological environment, belonging to rabbit embryos at the 8-cell and blastocyst stages, differentiated rabbit mammary epithelial cells during lactation, and differentiated cells of *Arabidopsis thaliana* plantlets. We developed new tools based on the processing of confocal images and a new statistical approach based on G- and F- distance functions used in spatial statistics. Our original computational scheme takes into account both size and shape variability by comparing, for each nucleus, the observed distribution against a reference distribution estimated by Monte-Carlo sampling over the same nucleus. This implicit normalization allowed similar data processing and extraction of rules in the five differentiated nuclei populations of the three studied biological systems, despite differences in chromosome number, genome organization and heterochromatin content. We showed that centromeres/chromocenters form significantly more regularly spaced patterns than expected under a completely random situation, suggesting that repulsive constraints or spatial inhomogeneities underlay the spatial organization of heterochromatic compartments. The proposed technique should be useful for identifying further spatial features in a wide range of cell types.

## Author Summary

Several reports suggest functional relationships within the spatial organization of the nucleus, gene regulation and cell differentiation. However, it still remains difficult to extract common rules, mostly because i) most data have been gathered on limited sets of nuclear elements and in nuclei outside their normal physiological environment, and ii) few three-dimensional (3D) quantitative measures have been performed. Thus, we questioned whether common nuclear organization principles exist in the animal and plant kingdoms. For that purpose, we investigated the 3D distribution of centromeres/chromocenters in five populations of animal and plant nuclei: rabbit embryos at 8-cell and blastocyst stages, rabbit mammary gland epithelial cells and *Arabidopsis thaliana* plantlets. We set up adapted procedures to segment confocal images and developed a new analytical methodology based on distances between positions within the nucleus and centromeres/chromocenters. We showed that in all systems, despite large differences in chromosome number (44 in rabbit; 10 in *A. thaliana*) and genome size (rabbit estimated size 2.77 Gbp; *A. thaliana* 125 Mbp), centromeres/chromocenters form significantly more regularly spaced patterns than expected under a completely random situation. This suggests that, whatever their specific features, conserved rules govern the spatial distribution of genomes in nuclei of differentiated cells.

## Introduction

In eukaryotes, the interphase nucleus is organized into distinct nuclear “compartments”, defined as macroscopic regions within the nucleus that are morphologically and/or functionally distinct from their surrounding [1]. Complex relationships between the spatial organization of these compartments and the regulation of genome function have been previously described. Furthermore, changes in nuclear architecture are among the most significant features of differentiation, development or malignant processes. Thus, these findings question whether topological landmarks and/or nuclear organization principles exist and, if so, whether these architectural principles are identical in the animal and plant kingdoms. To investigate nuclear organization principles, multidisciplinary approaches are required based on image analysis, computational biology and spatial statistics.

Spatial distributions of several compartments, which can be proteinaceous bodies or genomic domains, have been analyzed. Chromosome territories (CT), areas in which the genetic content of individual chromosomes are confined [2], [3], are usually radially distributed, with gene-rich chromosomes more centrally located than gene-poor chromosomes. Some studies report that chromosome size could also influence CT location [4]–[7]. Centromeres may be close to the nuclear periphery and those located on chromosomes bearing ribosomal genes are generally tethered to the nucleolar periphery [4]. Transcription sites, as well as early replicating foci, assumed to correspond to active chromatin, are more centrally located, whereas inactive heterochromatin tends to be at the nuclear periphery. At a finer level, active genes widely separated in *cis* or located on different chromosomes can colocalize to active transcription sites [8]–[10], whereas proximity to centromeric heterochromatin or to the nuclear periphery is generally associated with gene silencing [11]–[14]. Changes in the transcriptional status of genes have been frequently associated with their repositioning in the nucleus relative to their CTs, the nuclear periphery or the repressive centromeric heterochromatin [13], [15]–[20]. Furthermore, large reorganization in nuclear architecture (e.g. CTs, heterochromatic compartments, centromeres, speckles, nucleoli,..) can accompany some differentiation, development, malignant processes or natural variations [21]–[32].

However, it still remains difficult to extract common rules and establish comparisons due to various limitations. Indeed, most data have been gathered on limited sets of nuclear elements in isolated plant cell nuclei or in nuclei from immortalized animal cell lines outside their physiological environment, except for circulating blood cells. Little is known about possible differences in nuclear organization of cells within their tissue [33]. Some studies compared nuclear organization in primary cells versus cell lines, in cell lines versus tissues, and in 2D culture versus 3D cultures; these studies suggested that tissue architecture is involved in the control of nuclear organization [34]–[36]. In addition, data on nuclear organization in plant cell nuclei *in situ* are rare [37], [38]. Finally, few three-dimensional (3D) studies and quantitative measures have been performed to investigate spatial nuclear organization [39]–[42].

The statistics used to analyze the data were mostly based on radial patterns of nuclear elements, such as genes, chromosome territories, and centromeres. Radial positions have been measured with respect to the nuclear geometric center or the nuclear envelope [43], [44]. Spatial affinity between several elements has been investigated and spatial correlations have been assessed through central angles, for example between the radii joining homologous chromosome territory centers and the nuclear center [5], [45]. Alternative approaches based on distances between elements have been developed. Distances between a small number of elements, like two pairs of homologous alleles, were used for testing spatial attraction or repulsion [39]. Remarkably, spatial statistics tools, such as distance functions, that have been developed in ecology or epidemiology for analyzing spatial point patterns [46] have rarely been applied in nuclear organization studies. For example, (cross) nearest-neighbor distances have been used to analyze large numbers of nuclear elements, such as molecular complexes, PML bodies, or RNA Polymerase II foci [47], [48]. Alternatively, all pairwise inter-distances have been used to analyze the spatial distribution of chromocenters [49] and nucleocapsids [50].

In spatial statistics, data are usually collected through a sampling window over a single realization of a point process. This point process is generally considered as unbounded and spatially homogeneous. Such a theoretical framework makes sense in applications in which the investigated phenomenon extends far beyond the observed region. By contrast, analyses of nuclear spatial patterns are based on images of entire nuclei: the whole domain of interest is observed. Furthermore, one should not consider observed nuclear patterns as realizations of spatially homogeneous point processes.

Another difference is that replicated data are available as the analysis is carried out on a sample of nuclei. Recently, distance functions have been extended to replicated spatially heterogeneous point patterns [51], [52]. For instance, an extended F-function has been used for analyzing spatial patterns of transmissible spongiform encephalopathy lesions in brain tissue [53]. The extended F-function takes into account the expected spatial heterogeneity of the point process intensity. To estimate this intensity, the replicated patterns are first registered to locate all observed points in a common coordinate system. However, this type of preliminary registration is not possible for nuclei due to the lack of identifiable nuclear landmarks. Hence, further developments are required to make spatial statistics tools appropriate for nuclear spatial organization studies.

In this study, we develop an approach to furthering the analysis of nuclear spatial organization. Spatial distributions of nuclear compartments were quantified using the cumulative distribution functions of nearest-neighbor distances (G-function) and of distances between arbitrary points within the nucleus and their nearest compartment (F-function). The analysis of G- and F-functions was designed specifically to cope with patterns observed in non-registered and variable (both in size and shape) domains.

We applied this new approach to the investigation of the 3D distribution of centric/pericentric heterochromatin in five interphase nuclei populations belonging to the animal and plant kingdoms [54]. The centric/pericentric compartment was chosen due to its dual structural and regulatory functions. Indeed, it usually behaves as a transcription repressor and is essential for genome organization and the proper segregation of genetic information during cell division [55], [56]. This compartment often clusters and forms chromocenters [57]–[59]. We studied nuclei of cells at various differentiation stages, in three biological systems: rabbit embryos at the 8-cell and blastocyst stages, differentiated rabbit mammary epithelial cells during lactation, and differentiated cells of *A. thaliana* plantlets.

We found non-completely random and significantly more regularly spaced patterns than expected under complete randomness of the centric/pericentric heterochromatin compartment in the five differentiated cell populations, suggesting the existence of inter-kingdom nuclear organizational rules and possible nuclear regularities.

## Results

### Centric/pericentric heterochromatin markers

**Figure 1:**
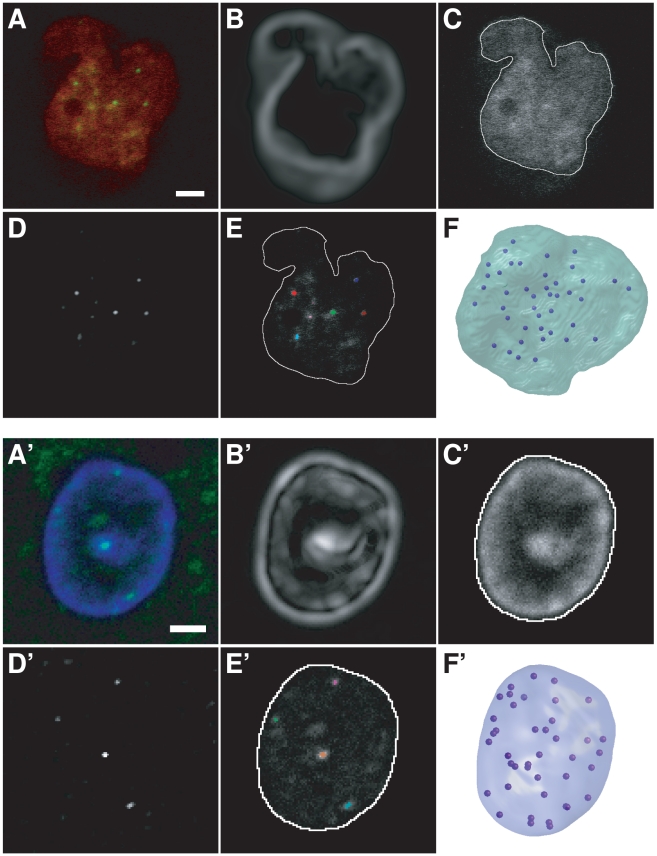
Image processing and 3D modeling of rabbit nuclei. (AA′) Single confocal microscopy sections. (A) Rabbit blastocyst nucleus, HP1β (red) and CENP (green) immunolabeling. (A′) Rabbit nucleus of a mammary gland epithelial cell, CENP (green) immunolabeling and DAPI counterstaining (blue). (BB′) Gaussian gradient magnitudes of nuclear staining images (HP1β or DAPI). (CC′) Overlay of nuclear contours (white) and DNA/HP1β staining. Contours were obtained after applying a Gaussian gradient-weighted threshold to nuclear staining images. (DD′) Enhancement of centromeric spots using top-hat filtering of CENP images. (EE′) Single section overlay of nuclear contours (white), CENP labeling, and centromeres (color) obtained by thresholding DD′. Note that centromeres are distributed within the whole nucleus, and not confined to the nuclear periphery. (FF′) Resulting 3D models with centromeres in dark blue. Scale bar: 2 µm.

The most common or comparable markers of the centromeres/chromocenters were chosen in the three biological systems, rabbit embryos, rabbit mammary gland and *A. thaliana*. The non-histone heterochromatin protein 1 (HP1β) family plays an important role in chromatin organization and transcriptional regulation of both heterochromatin and euchromatin compartments [60], [61]. Several HP1 isoforms are usually present in higher eukaryotes with various specificities and localization [60], [62]. The human or mouse HP1β isoform is usually used as marker for pericentric heterochromatin regions. However, our preliminary experiments revealed that immunodetection of HP1β in rabbit embryo, as well as in rabbit mammary gland nuclei, did not exhibit enough contrast to delineate the pericentromeric heterochromatin blocks (Figure 1A). By contrast, immunolabeling of centromeric proteins (CENP) using sera of patients with autoimmune diseases led to dots with significant differences in contrast, which allowed the positioning of the centromeres. HP1β–labeling was retained to label the whole nucleus in embryos.

In *A. thaliana*, LHP1, the HP1 homolog, is mainly involved in gene regulation and does not colocalize with centromeric heterochromatin [63], and therefore could not be used to follow heterochromatic centromeres. However, well-defined chromocenters can be revealed by DAPI staining in interphase nuclei, which mostly include centromeric and pericentromeric heterochromatic regions [57], [58].

**Figure 2:**
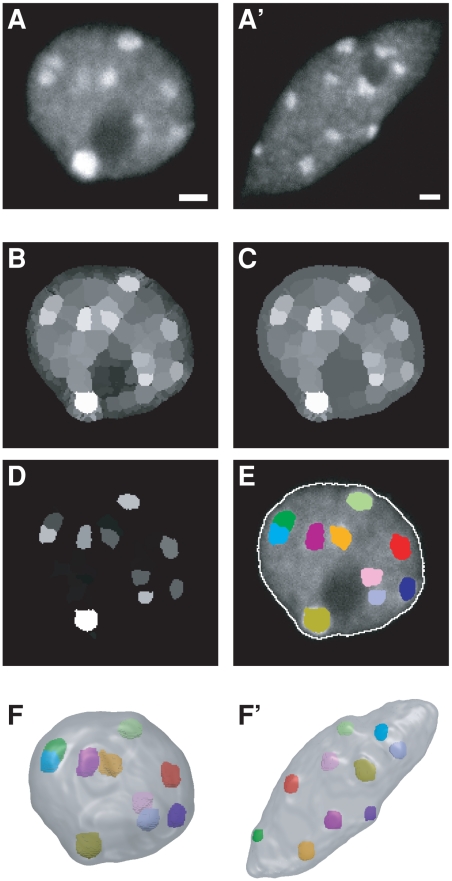
Image processing and 3D modeling of *Arabidopsis* nuclei. (AA′) Single confocal microscopy section through a rounded (A) and elongated (A′) nucleus. (B–E) Segmentation of the nuclear envelope and chromocenters. Following an initial partition of the 3D image using the watershed transform (B), a closing morphological operation was applied to the region adjacency graph (C) and each region was assigned a shape/contrast index (D). The chromocenters obtained by applying a threshold to the index map are shown in (E) as colored regions superimposed over the corresponding single section image. The nuclear boundary (white contour) was obtained by applying a threshold to the original 3D image. Note that chromocenters are distributed within the whole nucleus, and not confined to the nuclear periphery. (FF′) Resulting 3D models for the rounded (F) and elongated (F′) nuclei. Scale bar: 1 µm.

Therefore, nuclei of rabbit embryos, mammary gland and *A. thaliana* plantlets were labeled with CENP and HP1β, CENP and DAPI, and DAPI alone, respectively to visualize centromeres/chromocenters and the nuclear volume (Figures 1 and 2).

### Acquisition conditions and resulting image collection

After acquisition and treatment of a first set of images, capture conditions needed for a proper segmentation and for the best quality measurements were set up. We paid particular attention to i) the setup of the minimal (background) and maximal intensity level, ii) the spacing of the optical planes, and iii) the procedure to limit squeezing between slides and coverslips, particularly in the case of whole embryos. The acquisition parameters defined at this stage remained unchanged for the rest of the project. The resulting collection of images and the acquisition protocols have been deposited on the ICOPAN (“A 3D Image Collection of Plant and Animal Nuclei”) website (http://amib.jouy.inra.fr/icopan).

### Morphometric characterization of nuclei

Five populations of nuclei were analyzed. Nuclei were from rabbit embryos at the 8-cell and blastocyst stages, and from rabbit differentiated mammary epithelial cells (DMEC). DMEC nuclei were easily identified among nuclei of other mammary cell types based on their relative position within the tissue [64]. The DMEC flank the lumen of acini and are surrounded by elongated myoepithelial cells. Both cell types are buried within a stroma composed of adipocytes, fibroblasts and vascular cells. In *A. thaliana* plantlets, two populations of nuclei were analyzed based on their shapes: rounded or elongated nuclei (Figure 2 A and A′).

The size and shape parameters were determined for the five populations of nuclei and highlighted both a certain nuclear diversity between the various systems and homogeneity within each of them (Table 1, Figures 1 and 2). Shape analysis was detailed by determining flatness, compactness, and elongation indexes to characterize the 3D morphology of the studied nuclei. At the two rabbit embryonic developmental stages, the nuclei compactness value was rather low due to deep invaginations in the nuclear volume (Figure 1A). At the 8-cell stage, flatness was high (1.7, Table 1) and the main direction of flattening was closest to the Z-axis for almost all nuclei (28/29). The observed flattening may be due to the embryos being pressed between slides and coverslips. For blastocyst nuclei, the proportion of nuclei with the main direction of flattening close to the Z-axis was lower (25/41) and the flatness parameter for the 16 other nuclei was close to the overall average (1.36 vs 1.40). This suggested that, although experimental artifacts were partly responsible of the observed flattening, blastocyst nuclei were naturally relatively flat.

**Table 1:** Morphometric characterization of nuclei and number of detected centromeres/chromocenters.

Tissue	Cell type	n	Volume (µm^3^)	Compactness	Flatness	Elongation	Centromeres [CM] Chromocenters [CC]
Rabbit embryo	8-cell stage	29	1225 (346.1)	0.40 (0.11)	1.7 (0.3)	1.3 (0.2)	42.8 (1.8) [CM]
	Blastocyst stage	41	1140 (354.9)	0.58 (0.13)	1.4 (0.2)	1.2 (0.2)	43.7 (0.6) [CM]
Rabbit mammary gland	Epithelium	79	255 (28.4)	0.84 (0.07)	1.3 (0.2)	1.2 (0.1)	37.9 (2.4) [CM]
Arabidopsis plantlets	Round nuclei	59	83.4 (31.0)	0.78 (0.12)	1.5 (0.4)	1.1 (0.1)	8.0 (1.5) [CC]
	Elongated nuclei	58	182.7 (62.5)	0.41 (0.08)	1.7 (0.4)	2.6 (0.6)	10.9 (2.4) [CC]

For each cellular type, the table gives the number of analyzed nuclei (n) and the average size (Volume) and shape parameters (Compactness, flatness, and elongation), as well as the average numbers of detected centromeres (CM) or chromocenters (CC). Standard errors are given in parenthesis.

The observed DMEC nuclei were rather regular and spherical (high compactness and low flatness values). The main direction of flattening was closest to the Z-axis for most nuclei (57/79) suggesting that the low observed flattening may be partly experimental.

**Figure 3:**
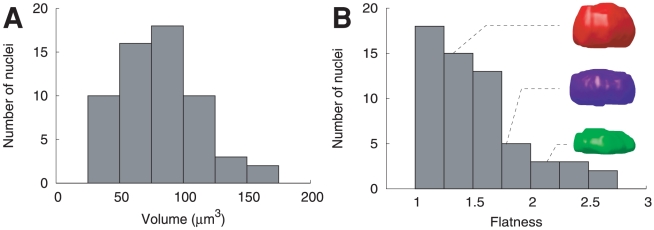
Distribution of size and shape parameters within the population of *Arabidopsis* rounded nuclei. (A) Histogram of nuclear volume. (B) Histogram of nuclear flatness (defined as the length ratio between the intermediate and the shortest nuclear axes). The sample 3D nuclear models illustrate low (red), moderate (blue), and high (green) flatness values.

The three rabbit nucleus populations showed unimodal distributions of volume, compactness and elongation, as expected in homogeneous populations. In *A. thaliana*, the nuclear volume within the population of rounded nuclei exhibited a unimodal distribution (Figure 3A), as did compactness and elongation (data not shown). Flatness distribution was also unimodal and was concentrated in the lower flatness range (Figure 3B). The distributions of the size and shape parameters thus confirmed that, though they were not selected based on cellular type, the rounded nuclei constitute a morphologically homogeneous population. Similar homogeneous distributions were observed within the population of elongated nuclei (data not shown). The main direction of flattening was closest to the Z-axis for 76% (45/59) of the rounded nuclei and flatness was close to 1 (i.e., no or moderate flattening) for the remaining nuclei. Similar observations were made within the population of elongated nuclei, in which 79% (48/58) of nuclei presented flattening oriented along the Z-axis. Thus, nuclear flatness measurements in the five analyzed populations suggested some experimental effects and a natural flatness in rabbit blastocyst nuclei.

### Detection of centromeres and chromocenters

Various segmentation procedures were developed to adapt to the size and contrast of the objects. Centromeres in rabbit embryo and mammary gland nuclei were revealed by CENP immunolabeling. In both cases, images were denoised with median and Gaussian filters, and the background lowered with a top-hat transform by size. Some of the CENP spots appeared to be outside of the nucleus mask, because of the elongation caused by the microscope's point spread function. To avoid truncating some of the spots, the nuclear masks were enlarged with a morphological dilation. Objects smaller than 0.02 µm^3^ were then removed in the masked CENP image.

In rabbit embryo, centromeres could not be extracted using a fixed threshold over all nuclei because of the high level of remaining background signal. Rather, the *a priori* knowledge of the number of centromeres (44) was used in searching for a threshold value that would produce, at most, 44 connected objects, starting with a threshold of 1 and incrementing by 1.

With this method, mean values of 42.8 and 43.7 centromeres were counted in rabbit 8-cell and blastocyst nuclei, respectively (Table 1). To assess the quality of the segmentation, subsamples of nuclei (6 at the 8-cell stage and 8 at the blastocyst stage) were checked visually. This revealed that at the 8-cell stage, 2.7% of segmented regions turned out not to be associated to HP1β labeling and thus not to be centromeric spots (false positives) and 4.7% of centromeric spots (as assessed by their association with HP1β labeling) were missed by the segmentation (false negatives). The false positive rate was 4.9% at the blastocyst stage, whereas no false negatives were identified. Finally, the centromeres were mapped within the 3D nucleus model (Figure 1F) for subsequent spatial analyses.

In rabbit mammary gland nuclei, a threshold computed as the median of the 11 brightest regional maxima divided by 4 was applied to each image. We identified a mean of about 38 centromeres per nucleus (Table 1). To visually check the result of the segmentation process, all input images were overlaid with their segmentations (Figure 1E′). Among 2996 segmented spots, 30 (1.0%) were considered to be false positives on the basis of their size or position. About 20 centromeres (0.7%) were under the threshold that had been set for intensity or size and were therefore not detected during segmentation (false negatives). The total number of visually detected centromeres was always lower than 44. Centromeres were mapped within the 3D nucleus model (Figure 1F′) for subsequent spatial analysis.

In *A. thaliana*, chromocenters could not be accurately detected via intensity thresholding. We thus developed an alternative strategy based on the fact that chromocenters have spherical or ellipsoidal shapes and present a positive contrast relative to their immediate neighborhood. Using a 3D watershed transform [65], the nucleus was partitioned into regions (Figure 2B). Each region was assigned a value given by the average intensity in the neighborhood of its barycenter. To correct for possible over-segmentation of chromocenters or nucleoli, region merging was repeatedly applied until all differences between values of adjacent regions were above a predefined threshold. The contrast of non-chromocenter regions adjacent to dark regions, such as the nucleolus, was reduced using a morphological region closing (Figure 2C). The contrast of each region was then computed as the average difference between its value and those of its neighbors, weighted by their sizes to limit the influence of small regions with exceptionally high or low values.

The contrast of each region was multiplied by its compactness to obtain a shape/contrast criterion that enhances chromocenters at the expense of other regions, even if they display similar intensities (Figure 2D). Using the ImageJ software [66], a threshold was then interactively set to a value ensuring the extraction of all chromocenters (Figure 2E). All segmentations were visually checked and compared to the original images by an experienced experimenter. Identified false positives were removed using the Free-D software [67]. Finally, the chromocenter regions were mapped within the 3D nucleus model (Figure 2 F and F′) for subsequent spatial analysis and their sizes quantified by their equivalent spherical diameters.

A few false negatives, generally corresponding to small and weakly labeled chromocenters that had been smoothed out when computing the Gaussian gradient, were also identified during the visual examination of segmented images. For rounded *A. thaliana* nuclei, the algorithm detected 470 chromocenters and the number of false negatives was 27 (error rate of 5.4%). For the elongated nuclei, 633 chromocenters were detected and 11 false negatives were identified (error rate of 1.7%).

The number of detected chromocenters differed between rounded and elongated nuclei (Table 1). Five to 10 chromocenters (average 8.0±1.5) were detected per nucleus in rounded nuclei. Our results therefore confirmed previously published data indicating that *A. thaliana* diploid cells (2n = 10) contain 4 to 10 chromocenters, due to a non-random association of homologous chromocenters or the coalescence of chromocenters containing rDNA repeats [58], [68]. Six to 17 (average 10.9±2.4) chromocenters were detected in elongated nuclei. Plants contain cell types with different ploidy levels that may vary from 2C (where 1C is the haploid genome complement) to 64C [69]. Previous studies reported a positive correlation between polyploidy and nuclear volume [38], [70]. Our data thus suggested that elongated nuclei, which on average contained more than 10 chromocenters and were ∼2 times larger than rounded nuclei (Table 1), were extracted, at least for a certain proportion of them, from endoreduplicated cells and that this population of nuclei may represent nuclei from cells that have undergone further differentiation.

### Non-completely random distribution of centromeres/chromocenters

Following the image processing stage, chromocenters and centromeres were segmented as regions within nuclei. To analyze their spatial distribution, all regions were represented by their centers of gravity, with, in the *A. thaliana* case, their equivalent spherical diameters. For the sake of brevity, we refer below to chromocenters/centromeres to mean their centers of gravity, with, in the *A. thaliana* case, their associated diameters.

Our method encompasses four key steps that can be summarized as follows and will be detailed below:

- The spatial patterns of centromeres/chromocenters within nuclei were quantified using two distance functions computed for each imaged nucleus.
- For each centromere/chromocenter pattern, observed distance functions were compared to the mean distance function associated with a completely random point pattern conditioned by the observed pattern size (number of points) and the observed nuclear space. In such a completely random binomial point process (CRBPP), points are distributed uniformly and independently [71]. For chromocenters, a variant of CRBPP was used involving a hardcore distance.
- Departures of observed distance functions from CRBPP mean distance functions were scored using p-values. Below, those scores will be referred to as spatial distribution indexes (SDI). Clustered patterns yield values close to 0; regular (evenly spaced) patterns yield values close to 1. The SDI of a CRBPP is uniformly distributed between 0 and 1. The computation of spatial indexes involves Monte-Carlo simulations.
- Statistical tests of departure from CRBPP were performed for each cell population using a goodness-of-fit test on SDI distributions.

**Figure 4:**
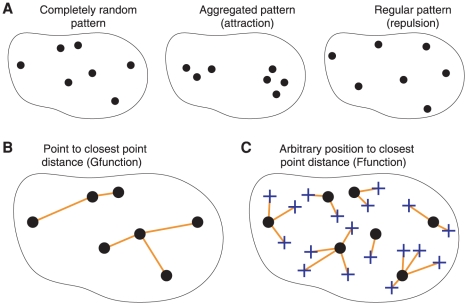
Spatial point patterns within the nucleus and distance functions. Centromere/chromocenter positions are represented as dots within nuclear contours. (A) Various types of spatial distribution. Positions can be uniformly and independently distributed (completely random pattern), or exhibit mutual attraction (aggregated pattern) or mutual repulsion (regular pattern). (B) The G-function is the cumulative distribution function of the distance between each centromere/chromocenter and its nearest neighbor (orange lines). This distance tends to be small for aggregated patterns and large for regular patterns. (C) The F-function is the cumulative distribution function of the distance between typical nuclear positions (blue crosses) and their nearest centromere/chromocenter (orange lines). This distance tends to be large for aggregated patterns and small for regular patterns.

Distance functions are standard tools in the statistical analysis of spatial point processes [46]. The nearest neighbor distance function G of a point pattern is the cumulative distribution function of the distance *X* between a typical point (i.e., a uniformly randomly chosen point) of the pattern and its nearest neighbor (Figure 4):
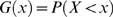
Computing this function from the point pattern is straightforward. The F-function is the cumulative distribution function of the distance Y between a typical position within the nucleus and its closest point in the pattern:
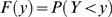
Thus, F(y) is the nuclear volume fraction that lies at a distance less than y from a point of the pattern. We are considering the distribution of centromeres/chromocenters as a finite process within the bounded nuclear space. To analyze populations of nuclei, we developed an original strategy, based on F- and G-functions, which does not require nucleus registration.

A stochastic scheme was adopted to compute the F-function corresponding to a nuclear point pattern. A number of independent evaluation points N_E_ were generated uniformly at random within the nucleus. For each evaluation point, the distance to the closest point of the pattern was determined (Figure 4). The cumulative distribution function F(y) was then estimated by the proportion of evaluation points for which this distance is below y. Setting N_E_ to 10000 was sufficient to smooth out the effect of evaluation point sampling on the F-function.

To determine whether the spatial distribution of centromeres/chromocenters obeys any organizational rule, the observed distributions were compared against a completely random distribution, conditioned on the observed numbers of centromeres/chromocenters and, in *A. thaliana*, on chromocenter sizes. Due to the arbitrary shape of the nucleus, the expected distance functions under CRBPP cannot be determined analytically. A Monte-Carlo approach was therefore adopted, whereby the distance functions were computed over sets of patterns simulated according to CRBPP. For each nucleus, random patterns were generated with the same number of centromeres/chromocenters as detected within the nucleus. In *A. thaliana*, each random point was also assigned the radius of one chromocenter. This hardcore distance defined a sphere within which no other point was allowed to fall and a minimum distance between the point and the nuclear envelope. Taking care of rabbit centromere sizes was not necessary because of their small size. The CRBPP distance functions were estimated by computing averages over a number P1 = 500 of such independent patterns.

**Figure 5:**
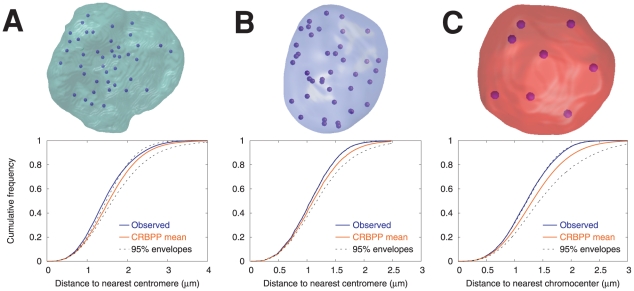
Sample observed and CRBPP estimated mean F-functions. (A) Rabbit blastocyst nucleus (same as in Fig. 1A). (B) Rabbit mammary gland nucleus (same as in Fig. 1A′). (C) *Arabidopsis* rounded nucleus (same as in Fig. 2A). In each case, a 3D model of the centromere/chromocenter positions within the nuclear envelope is displayed above the corresponding observed F-function (blue), the CRBPP mean F-function estimated by Monte-Carlo simulations (orange), and the 95% envelope (dotted black).

Observed and CRBPP mean F-functions obtained for the three nuclei from Figures 1A, 1A′ and 2A are displayed in Figure 5 (A: rabbit embryo; B: rabbit mammary gland; C: *A. thaliana* leaflets). As illustrated by these examples, the observed F-functions were frequently located on the left side of the CRBPP ones, and presented a steeper slope. On average, the observed distance between any nuclear position and the closest centromere/chromocenter was thus lower and less variable than expected under CRBPP. This suggests a non-completely random and regular distribution of centromeres/chromocenters within the nucleus (see Figure 4). Discerning any particular trend by visually examining G-functions was much more difficult (data not shown). This may be due to the fact that i) this function is potentially less discriminant than the F-function and the fact that ii) it was estimated from a smaller pool of data (number of detected centromeres or chromocenters compared with an arbitrary number of arbitrary positions for F-function).

The next step in our analysis was to test, at the population level, the statistical significance of the differences between the observed F- and G-functions and the CRBPP theoretical F- and G- functions. Due to the arbitrary shape of the nucleus, the fluctuations under CRBPP of the distance functions around their averages are not analytically accessible and a Monte-Carlo approach was designed. To avoid under-estimation of these variations, they were estimated using a second set of randomly generated P2 = 500 patterns. For each simulated pattern, the difference between its distance function and the CRBPP theoretical function was defined as the signed difference of maximum amplitude. Taking for example the F-function, we thus have:
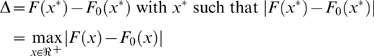
where F_0_ is the F-function under CRBPP computed using the Monte-Carlo approach described above.

We also computed the difference between the observed pattern distance function and that of the CRBPP; this yielded a total of P2+1 differences. A p-value (the probability of observing, under CRBPP, a difference at least as large as that observed) could then be computed for each nucleus as the proportion of random patterns with a difference equal to or above that observed. Since it quantifies a spatial repartition, this p-value was called spatial distribution index (SDI). For example, low values of the SDI associated to the F-function indicate regularity in the patterns (evenly distributed points) while high values correspond to clustered patterns. Under the hypothesis that centromeres/chromocenters obey CRBPP, the SDI within a population is uniformly distributed between 0 and 1. Our test thus consists in comparing the observed SDI distribution with the uniform distribution. This was done using the two-sided Kolmogorov-Smirnov test (α = 5%) in the R statistical software package [72].

**Figure 6:**
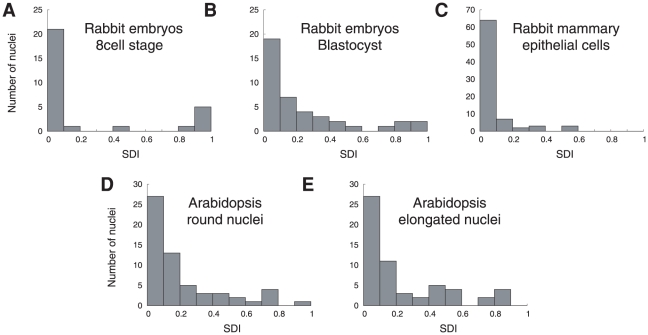
Histograms of the F-function-related spatial distribution index, within the five cellular types. For each nucleus, the SDI is the probability of observing, under a completely random binomial point pattern, a difference at least as large as that observed between the empirical and the CRBPP mean F-functions.

Within the five groups, the distributions of the SDI based on F-function were significantly different from the uniform distribution (Figure 6 and Table 2). Hence, the spatial distributions of centromeres and chromocenters are different from the completely random distribution. Besides, the histograms were concentrated in the lower range of SDI (Figure 6), meaning that the observed F-functions were generally above the CRBPP ones. This analysis thus demonstrated that the centromeres or chromocenters tend to form more regularly spaced patterns than expected under CRBPP. For G-functions, the distributions of the SDI within the five groups (data not shown) were also significantly different from the uniform distribution (Table 2). The SDI histograms were concentrated in the upper range, meaning that the observed G-functions were generally below the CRBPP ones and that the nearest centromere/chromocenter was on average farther away than expected under CRBPP. Thus, though departure from complete randomness was less pronounced, analysis by G-function was consistent with the results obtained with F-function.

**Table 2:** Analysis of the spatial distribution of centromere/chromocenter: results of statistical tests.

			F-function		G-function		Correlation with flatness	
Tissue	Cell type	n	D	p-value	D	p-value	tau	p-value
Rabbit embryo	8-cell stage	29	0.62	3.0 10^−10^	0.43	3.7 10^−5^	−0.27	0.04
	Blastocyst stage	41	0.50	3.4 10^−9^	0.34	1.2 10^−4^	−0.10	0.48
Rabbit mammary gland	Epithelium	79	0.74	<2.2 10^−16^	0.42	2.4 10^−12^	−0.24	0.01
Arabidopsis plantlets	Round nuclei	59	0.49	1.0 10^−12^	0.26	7.2 10^−4^	0.02	0.88
	Elongated nuclei	58	0.46	3.9 10^−11^	0.36	5.8 10^−7^	−0.04	0.69

For F- and G-functions, the table gives the statistic (D) and the p-value of the bilateral Kolmogorov-Smirnov test that was used to assess differences between observed distributions and completely random patterns. The table also gives the statistic (tau) and the p-value of the Kendall's rank correlation test that was used to assess a link between observed centromere/chromocenter spatial distributions and nucleus flatness. n: number of analyzed nuclei.

Lastly, we examined whether the regularity of the spatial distribution of centromeres or chromocenters could be explained by the experimental flattening of the nuclei along the Z direction. A link between flatness and SDI was tested on the nuclei with a Z-oriented minor axis. As flatness (e.g., Figure 3B) and rank (Figure 6) distributions largely deviate from normality, a non-parametric test based on Kendall's tau rank correlation [73] was applied (α = 5%). In *A. thaliana* (rounded and elongated nuclei) and blastocyst nuclei, no correlation was found between flatness and the F-function-associated SDI (Table 2). In 8-cell embryo and mammary gland nuclei, correlations between flatness and F-function SDI turned out to be significant (Table 2). However, the values of tau remained rather small (about 30%). Therefore, even for those two latter cell populations, the regularity of centromere patterns cannot be considered as caused by flattening. Similarly, no relation was found between nucleus flatness and the SDI associated with G-function. Consequently, the possibility that flattening along the Z direction is responsible for the regular spatial distribution of centromeres/chromocenters was ruled out.

## Discussion

The Rabl configuration observed in salamander [74], fission yeast [75], drosophila embryos [76], as well as in some plants such as *Allium cepia* [77], wheat, rye, barley and oats [78], [79] but not in *A. thaliana* nor in mammals, is probably the most spectacular example of a non-random organization in interphase nuclei. In this configuration, the centromeres are clustered at one end of the nucleus and telomeres at the opposite end. Besides this extremely recognizable organization, detection of any regularity in the complex nuclear organization is difficult and requires the development of specific 3D image analysis and statistical tools. Indeed, as nuclei exhibit large morphological fluctuations both within and between cellular types [80], spatial normalization is required. However, no nuclear landmark has yet been identified as a suitable reference to perform this standardization [40]. In this study, we addressed this issue and developed original tools adapted to various biological models, allowing a common treatment of data and extraction of rules. Building on well-established distance functions from spatial statistics, we designed a new methodological framework to analyze replicated samples without explicit nucleus registration.

Spatial statistics distance functions have only rarely been applied to study nuclear organizations. Nearest neighbor distances (G-function) have been used to characterize the spatial distribution of transcription factors [47], [48], [81], centromeres [49], and other nuclear compartments [82]. The distribution of all inter-distances (quantified through the pair correlation function or the K- and L-functions) has also been considered [49], [81]. To our knowledge, the present study is the first to rely on the F-function (the cumulative distribution of the distance from arbitrary nuclear positions to the nearest centromere/chromocenter) to investigate nuclear architecture. In contrast to the F-function, the G, K and L-functions and the pair correlation function only depend on the relative positioning of the points within the pattern, irrespective of their absolute positioning within the nucleus. For instance, a translation of a clustered pattern from the nucleus center to its periphery has no effect on the G-function while it will tend to shift the F-function towards the left (larger empty nuclear region). Thus, in the finite and bounded context of nuclear organization studies, the F-function captures more information about spatial repartition and therefore represents a potentially more discriminant and sensitive tool to detect differences between spatial distributions. In accordance with this, our results showed more pronounced departure from complete randomness with F-function than with G-function, be it in individual function plots or SDI distributions. The J-function, a more recently introduced function [83], combines the F- and G-functions and has an easy interpretation for point patterns with random size. However, it needs to be further elaborated for analyses conditioned on the number of points.

The completely random patterns that were simulated here contained a number of points equal to that actually observed in the patterns and not to the number of chromosomes in the species. In *A. thaliana*, it is known that centromeres aggregate into a variable number of chromocenters. In the rabbit, exactly 44 centromeres have never been visually observed in interphase nuclei, be it because of aggregation, limited optical resolution, or labeling issues. Hence, the number of chromosomes only provides an upper bound on the expected number of points. For this reason, our analysis has focused on the spatial distribution of *observed* patterns.

The F-function was computed using a Monte-Carlo scheme for sampling the nuclear space. An alternative computational scheme to estimate the F-function consists in computing the Euclidean distance map (EDM) [84], [85], which gives the distance between each voxel of the nucleus and the closest voxel containing a centromere or chromocenter center of gravity; the F-function is then given by the normalized cumulative histogram of the distance map. In our preliminary experiments, we have implemented, tested, and compared the two approaches. We have retained the estimation of F based on randomly generated points throughout the nucleus for essentially two reasons. The first one is that this approach retains the subpixel precision at which the positions (centers of mass) of centromeres and chromocenters are computed. On the contrary, using distance maps introduces a loss of precision because these positions must be rounded to voxel (integer) coordinates. This is not critical in our case because the voxel size is small as compared to the nucleus size. However, this effect could bias the analysis when processing data extracted from images with large voxel sizes. The second reason is computational efficiency: our experience did not reveal any computational advantage of the EDM approach over the stochastic one.

Standard methods for computing empirical G- and F-functions usually include boundary corrections [86]. Since point patterns are traditionally observed within sampling windows, nearest point distances can indeed be over-estimated due to the exclusion of some points from the experiment. For the G-function, for example, the Hanisch correction [87] consists in discarding the recorded points whose nearest neighbor is farther than the boundary. Such standard estimation corrections have been applied in studies on nuclear patterns [49], [81]. In this context, however, no point (here, centromere or chromocenter) is expected outside the nucleus. Hence, edge-corrections should be all the more avoided. Indeed they may decrease statistical power by reducing the number of analyzed points and potentially bias the analysis, especially if the assumed sampling window is computed from the pattern itself [49]. To obtain unbiased estimates of G- and F-functions, we proposed an alternative computational scheme that takes into account the actual boundary of the nucleus and involves no edge-correction.

Previous spatial statistical analyses of populations of nuclei have been conducted based on distance functions either averaged without normalization [49], [81] or pooled after standardization with respect to the greatest inter-object distance, to account for nuclear size fluctuations [47]. This left the difficult issue of nuclear shape normalization unsolved. A first significant contribution of the approach described here is to take into account both size and shape variability. This was achieved by comparing, for each nucleus, the observed distribution against a reference distribution estimated by Monte-Carlo sampling within the same nucleus. This implicit normalization (each nucleus being its own reference) circumvents the unfeasibility of an explicit nucleus registration in the absence of identified nuclear reference points [40]. A second significant contribution of the present study is the introduction of a test for complete randomness at the population level. For each nucleus, the departure from the completely random spatial distribution was quantified through a spatial distribution index (SDI). This SDI could have been used to independently classify each centromere or chromocenter pattern as completely random (CRBPP) or not. Then a global conclusion concerning the population level could have been drawn by a simple proportion test. However, by such a binarization of the SDIs, one focuses mainly on extreme patterns (clustered or regular) and may fail to detect slight deviations from complete randomness. Avoiding binarization gives more sensitivity to detect spatial structure.

Overall, our methodology therefore allows for sensitive and unbiased statistical assessment of distribution differences against reference distributions. Using this approach, we showed that, in three biological systems belonging to plant and animal kingdoms and in the five types of interphase nuclei, centromeres/chromocenters form significantly more regularly spaced patterns than expected under a complete random situation. Interestingly, this feature was found in biological systems with extremely different numbers of chromosomes (44 in rabbit versus 10 in *A. thaliana*) and different genome sizes (rabbit estimated size 2.77 Gbp; J. Johnson, Broad Institute, personal communication [https://www.broadinstitute.org/ftp/pub/assemblies/mammals/rabbit/oryCun2/Stats.pdf]; *A. thaliana* last estimated size 125 Mbp [http://arabidopsis.org/portals/genAnnotation/], [88]). This suggests that conserved rules govern the spatial distribution of genomes, whatever their specific features.

In *A. thaliana*, 2D analyses suggested non-uniform distributions of some nuclear elements, such as telomeres, mostly localized in the vicinity of the nucleolus [89], as well as the non-random association of homologous chromocenters or chromocenters that contain homologous rDNA repeats [58]. More recent studies based on 3D imaging suggested the existence of a radial arrangement pattern in *A. thaliana* interphase nuclei, with centromeres localized predominantly at the nuclear periphery [43], [90]. Our results are consistent with a non-random distribution of *A. thaliana* heterochromatic domains, but further demonstrate their tendency to form regular patterns. The prevailing hypothesis about the spatial organization of *A. thaliana* interphase nuclei is that of a globally random distribution of chromosome territories under non-specific constraints and interactions [2], [90], [91]. According to this view, the regular distribution of chromocenters we have observed could merely result from the partitioning of the nucleus into distinct chromosome territories. Whether more specific mechanisms of mutual repulsion exist remains to be elucidated, but in any case, the concomitant aggregation of some centromeres into chromocenters and the regular distribution of chromocenters suggest that constraints or control mechanisms are exerted at multiple scales to determine the positioning of heterochromatic domains in *A. thaliana* nuclei.

A priori, a SDI distribution may differ from CRBPP due to spatial heterogeneity or due to spatial interactions (or both). The volume occupied by the nucleoli, which can be rather large in some biological systems (rabbit), are sources of heterogeneity that have not been taken into account in our analyses since simulated points could fall anywhere within the nucleus. However, the only bias that may have resulted from neglecting these excluded nuclear regions is a bias toward artifact aggregation. Preliminary calculations, performed on a subset of rabbit nuclei, showed that taking into account the nucleolus volume accentuated the deviation from the CRBPP (data not shown). This confirmed that the observed regular dispersion of centromeres/chromocenters was not due to nucleoli volume omission. Nevertheless, further developments will be necessary to integrate the nucleoli into the 3D nuclear modeling and analyses. Other spatial inhomogeneities, due for example to the size and nuclear position of the chromosome territories, may also impact on the distribution of centromeres/chromocenters, especially in species with a large range of chromosome sizes and shapes (e.g. rabbit) [92]. It would therefore be interesting to refine the model by taking into account, whenever possible, the identity of all or most of centromeres/chromocenters. In rabbit, probes allowing the detection of four centromeric DNA families are already available [93].

Previous authors have reported clustering of pericentric heterochromatin during terminal differentiation, e.g. during myogenenesis [94], in human neutrophils [28], human and mouse neurons [95], [96] and rat myoblasts [97]. In these biological models, centromeres cluster into chromocenters and comparisons between undifferentiated and differentiated cell nuclei show that the number of chromocenters decreases during the differentiation process. These results may seem contradictory with the regular patterns revealed by the F-function, especially in *A. thaliana* in which chromocenters are also observed. However, our analysis concerned the spatial distribution of chromocenters instead of their number. In particular, each given observed pattern of chromocenters was compared with simulated random patterns of the same size. Results showed that the observed patterns are more regularly spaced than completely random patterns of the same size. Therefore, it appears that centromeres in *A. thaliana* differentiated cells cluster into chromocenters that are regularly spaced within the nucleus. In differentiated rabbit cells, centromeres do not form chromocenters, but the numbers of detected centromeres in mammary gland cells indicate that a small fraction of centromeres may cluster. In the rabbit mammary gland model, centromeres and a few small clusters of centromeres are more regularly spaced than completely random patterns.

The approach and the tools we developed could now be applied to other nuclear compartments and -even more interestingly- to other differentiation stages, to determine whether this tendency to form more regularly spaced patterns than expected under complete randomness might represent a “signature” of the differentiated states. This methodology can also be generalized to investigate further the properties of intra-nuclear spatial distributions. Indeed, our strategy consists in comparing, on the basis of appropriate distance functions, observed nuclear organizations to patterns simulated from a reference distribution. Introducing constraints on the simulated point patterns, as for example interactions with the nuclear envelope or other nuclear compartments, will allow evaluating further hypotheses beyond randomness.

## Materials and Methods

### Ethics statement

All experiments involving animals were carried out according to European regulations on animal welfare.

### Biological materials

All animals were handled following ethical rules for animal welfare according to the INRA ethics statement. Rabbit embryos were obtained by natural fertilization of superovulated mature New Zealand White rabbit females, as already described [98], [99]. Superovulation was induced by two 0.25 mg, two 0.65 mg and one 0.25 mg intramuscular injections of follicle-stimulating hormone (FSH, Stimufol, Mérial, Lyon, France) given 12 hours apart. Females were mated with males 12 hours after the last FSH injection and 30 IU of human chorionic gonadotropin (hCG, Choluron, Intervet, Angers, France) was injected a few minutes after mating. In rabbit, fertilization occurred at ∼12 hours post coitum (hpc). Two-cell embryos were collected in the rabbit oviduct at 24 hpc and were further cultured *in vitro* in B2 medium supplemented with 2.5% fetal calf serum (Sigma) in 5% CO_2_ atmosphere at 38.5°C until the 8-cell (48 hpc) and blastocyst (100 hpc) stages.

Mammary glands were collected from 16-day lactating New Zealand INRA-1077 rabbits, one hour after suckling. Mammary gland tissues were cut into small pieces, fixed in 4% paraformaldehyde (PFA) in phosphate-buffered saline (PBS) for 30 min at room temperature (RT), washed three times with PBS and equilibrated in 40% sucrose before embedding in Cryomount (Histolab) and snap freezing in liquid nitrogen. Samples were conserved at −80°C. Cryosections of about 14 µm in thickness were prepared on a Reichert Jung cryo-microtome (Leica, Wetzlar, Germany), deposited on slides (SuperFrost Plus glass slides, Menzel-Gläser J1800AMNZ) and stored at −80°C until use.

*A. thaliana* plantlets (Col-0 accession) were grown *in vitro* as previously described [25]. Three-week-old plantlets were fixed for 30 min in 4% PFA in PBS buffer (PFA-PBS), under vacuum, at room temperature. The fixative was replaced, and plantlets were fixed for an additional 30 min in the same conditions. Up to 8 fixed seedlings were transferred into an Eppendorf tube and gently ground in 500 µl of extraction buffer (10 mM Tris HCl pH 7, 4 mM spermidine, 1 mM spermine, 5 mM MgCl2, 0.1% triton X-100, 5 mM β-mercaptoethanol). Nuclei suspension was filtered through a 50 µm nylon mesh. After gentle centrifugation (500× g, 3 min), the pellet was washed in 1× PBS, treated with 0.5% triton X-100 in PBS and washed in PBS. Nuclei were resuspended in 30 µl PBS.

### Immunoprocessing and labeling

Rabbit embryos at 8-cell and blastocyst stages were fixed overnight at 4°C in 4% PFA-PBS, permeabilized 30 min at RT with 0.5% Triton X-100, and blocked with 3% bovine serum albumin in PBS (BSA-PBS) for 1 hour [30].

Fixed mammary gland sections were incubated in 50 mM NH_4_Cl in PBS for 15 min and washed with PBS. They were then permeabilized with 0.5% Triton X-100 for 30 min at RT, washed again with PBS, and blocked with 2% BSA-PBS for 1 hour at RT.

Immunoprocessing was then similar for rabbit embryos and mammary gland sections. Fixed embryos and slides with fixed mammary gland sections were incubated with the primary antibodies overnight at 4°C in 2% BSA-PBS. After three washes with 0.05% or 0.1% tween-20 in PBS at RT (15 min each), incubation with the secondary antibodies was performed for 1 hour in 2% BSA-PBS at RT followed by three washes (10 min each) with 0.1% tween-20 in PBS at RT. For double immunostaining, primary antibodies and secondary antibodies were mixed together at the same final dilutions as for simple immunodetection. Rabbit embryos were then deposited on slides and mounted in VECTASHIELD medium (Vector laboratories, Burlingame, CA). Mammary gland sections were washed once in PBS, counterstained with DAPI (1 µg/ml in PBS for 5 min, at RT), washed in PBS for 5 min at RT and mounted under a coverslip with ProLong Gold antifade reagent (Invitrogen).

The suspension of *A. thaliana* nuclei was spotted on a slide and left to evaporate at 4°C for 20 min, before mounting in VECTASHIELD medium with 1 µg/µl of DAPI for DNA counterstaining.

### Antibodies

HP1β was detected with a mouse monoclonal anti-HP1β antibody (clone 1 MOD 1A9, dilution 1∶250), and revealed with a lissamine–rhodamine-conjugated anti-mouse secondary antibody (Jackson ImmunoResearch, dilution 1∶150). Centromeres were detected in rabbit embryos and in rabbit mammary glands by a human autoantibody against centromeres (HCT-0100, Immunovision, dilution1∶300) followed by FITC-conjugated donkey anti-human antibody (Jackson ImmunoResearch, dilutions 1∶150 in rabbit embryos and 1∶300 in rabbit mammary glands).

### Imaging

Embryos were scanned with a Zeiss LSM 510 confocal laser-scanning microscope equipped with a ×63/NA 1.4 oil immersion objective. Z stack images were acquired at intervals of 0.24 µm with 488-, 543- and 633-nm wavelengths of the lasers and with an XY voxel size of 0.04 µm.

Images of mammary gland sections were captured with an optical sectioning microscope attached to an AxioObserver imaging Apotome system (Zeiss) (×63/NA 1.4 oil immersion objective). Z stack images were acquired at intervals of 0.24 µm on two channels (DAPI and FITC), with an XY voxel size of 0.1 µm.

*A. thaliana* nuclei images were captured on a Leica DM IRE2 SP2 AOBS spectral confocal microscope equipped with a 405 nm diode (25 mW) using a ×63 HCX PL APO objective (NA 1.2). Z stack images were acquired at intervals of 0.122 µm, with an XY voxel size of 0.05 µm.

The anisotropy of voxel sizes in XY-Z was taken into account in all subsequent image processing and spatial analysis procedures.

Images can be freely retrieved in their native formats together with detailed acquisition protocols at the ICOPAN Website (http://amib.jouy.inra.fr/icopan).

### Definition of the nuclear periphery from HP1β and DAPI labeling

Nucleus contours were determined on the HP1β (embryos) and DAPI (mammary gland and *A. thaliana*) images.

Images of rabbit nuclei were denoised with a median filter and a Gaussian filter. They were subsequently segmented through two different pathways.

HP1β images (embryos), on which several nuclei are present, were analyzed with the Insight Toolkit (ITK) library. The robust automatic threshold selection method (RATS) [100] was used to compute a threshold to ‘binarize’ the HP1β images. The threshold is computed as the mean of the intensity values in the HP1β image weighted by their Gaussian gradient magnitude. To avoid the high gradient values in the nucleus caused by non-homogeneous content, the small bright and dark zones were removed with a 3D area opening and a grayscale fill hole transformation before computing the gradient. The joined masks of nuclei were separated using a watershed transform on the distance map. Truncated nuclei at the image border as well as objects smaller than 200 µm^3^ were removed.

A semi-automated procedure was developed to segment mammary gland nuclei from the DAPI image. DAPI signal was denoised with a median and a Gaussian filter, and manually thresholded to produce partial nuclear masks. The DAPI signal was mostly present on the border of the nuclei. As a result, thresholding this signal results in an incomplete nucleus, in which the center is not filled and the border is not continuous. The nuclear borders were thus closed with a morphological closing transform with a large round kernel, and content of the nuclei was filled with a binary hole filling transform. The masks of the different nuclei were then separated by a watershed transform on the distance map, and the nuclei from the cell types of interest were manually selected.

Confocal image stacks of *A. thaliana* nuclei were processed and analyzed with programs developed using the Free-D software libraries [67]. Each image stack contained a single nucleus. Images were automatically cropped to limit processing to a bounding box surrounding the nucleus. To separate the nucleus from the background, a preliminary intensity threshold was then computed using the isodata algorithm [101]. This algorithm is sensitive to the relative size of the nucleus within the image. As a result, the threshold was generally too high because of the larger background size. To correct for this bias, the intensity average m and standard-deviation s were computed over the nucleus region defined by the preliminary threshold and the actual threshold was set to m-2s. The resulting binary image generally contained holes, corresponding in particular to the nucleolus, and presented boundary irregularities due to noise. In addition, bumps were also observed on some nuclei at their basal and apical faces, because of blur from chromocenters [39]. Hole filling, opening and closing binary morphological operators [65] were therefore applied to regularize the binary image. The subsequent processing and analyses were confined to this final nucleus mask. A surface model of the nuclear envelope was generated by applying the marching cubes algorithm [102] to the binary mask.

### Morphometric analysis of nuclei

Nuclear size was quantified by nuclear volume. Nuclear shape was quantified using the compactness parameter, which is given by:
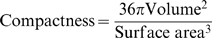
This parameter takes its maximum value 1.0 for a sphere and decreases toward 0.0 as the shape surface becomes less regular.

Visual image examination revealed that some nuclei were flattened along the Z-axis; thus, a flatness parameter was defined, based on the lengths of the principal axes of the nuclear surface:
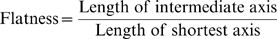
Symmetrically, an elongation parameter was also defined:
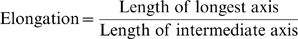
The main direction of flattening (resp. elongation) was defined by the coordinate frame axis that was the closest to the shortest (resp. the longest) principal axis of the nucleus.
